# Timing of global regression and microbial bloom linked with the Permian-Triassic boundary mass extinction: implications for driving mechanisms

**DOI:** 10.1038/srep43630

**Published:** 2017-03-06

**Authors:** Björn Baresel, Hugo Bucher, Borhan Bagherpour, Morgane Brosse, Kuang Guodun, Urs Schaltegger

**Affiliations:** 1 Department of Earth Sciences, University of Geneva, Rue des Maraîchers 13, 1205 Geneva, Switzerland; 2 Paleontological Institute and Museum, University of Zurich, Karl Schmid-Strasse 4, 8006 Zurich, Switzerland; 3 Guangxi Bureau of Geology and Mineral Resources, Jiangzheng Road 1, 530023 Nanning, China

## Abstract

New high-resolution U-Pb dates indicate a duration of 89 ± 38 kyr for the Permian hiatus and of 14 ± 57 kyr for the overlying Triassic microbial limestone in shallow water settings of the Nanpanjiang Basin, South China. The age and duration of the hiatus coincides with the Permian-Triassic boundary (PTB) and the extinction interval in the Meishan Global Stratotype Section and Point, and strongly supports a glacio-eustatic regression, which best explains the genesis of the worldwide hiatus straddling the PTB in shallow water records. In adjacent deep marine troughs, rates of sediment accumulation display a six-fold decrease across the PTB compatible with a dryer and cooler climate as indicated by terrestrial plants. Our model of the Permian-Triassic boundary mass extinction (PTBME) hinges on the synchronicity of the hiatus with the onset of the Siberian Traps volcanism. This early eruptive phase released sulfur-rich volatiles into the stratosphere, thus simultaneously eliciting a short-lived ice age responsible for the global regression and a brief but intense acidification. Abrupt cooling, shrunk habitats on shelves and acidification may all have synergistically triggered the PTBME. Subsequently, the build-up of volcanic CO_2_ induced a transient cool climate whose early phase saw the deposition of the microbial limestone.

Since the early days of stratigraphy, mass extinctions were noticed to coincide with major and global sea-level changes^1^,^2^ that significantly alter extinction patterns and time-series of geochemical proxies. In the case of the Permian-Triassic boundary mass extinction (PTBME), the system boundary itself has been initially placed during a global eustatic regression^3^, but was subsequently placed during a global transgression^4^. The sea-level rise scenario naturally paved the way for a concomitant rise of the oxygen minimum zone as a kill mechanism, to which “lethally” hot sea-surface temperatures were recently added^5^,^6^. With the exceptions of deep oceanic settings (e.g., Arrow Rocks, New Zealand^7^; Mino Tamba Terrane, Japan^8^,^9^), of few deep water shelves (e.g., Dongpan, South China^10^) and of extremely rare shallow shelves (Musandam, United Arab Emirates^11^), an overwhelming majority of globally distributed Permian-Triassic boundary (PTB) sections are affected by an unconformity separating the last Permian rocks from the first Triassic ones (e.g., Armenia^12^; Canada^13^; Pakistan^14^; Norway^15^; China^16^). Intensively studied shallow water sections from South China follow this rule and the origin of this hiatus is intensively debated. Two competing mechanisms are frequently proposed: submarine dissolution through acidified waters^17^ or subaerial erosion^18^. However, both mechanisms are not mutually exclusive and may conceivably have acted simultaneously^19^. In this respect, we emphasize here that climate-driven eustatic sea-level changes are commonly at the decamillennial timescale, an order of magnitude compatible with the duration of the world-wide regression that coincides with the PTB. The base of the Triassic in the equatorial shallow water sections is usually represented by the iconic microbial limestone^16^, which has been interpreted as the hallmark of ecosystem devastation in the immediate aftermath of the PTBME. This “devastated” view is questioned by the recent discovery of diversified Triassic (Griesbachian) shelly faunas that mechanically accumulated between domical microbialites^20^,^21^,^22^. Although carbonate super-saturation, warm waters and absence of clastic input were all prerequisites for the development of microbial limestone in the >12000 km^2^ Luolou Platform of the Nanpanjiang Basin^19^, how to switch swiftly from acidic to alkaline and CaCO_3_ super-saturated waters remains open. However, a sudden increase of accommodation space leading to the burial of microbialite-bearing fault bounded blocks under siliciclastic sediments is the most likely explanation for the abrupt cessation of the microbial limestone in the Nanpanjiang Basin (South China). No evidence in support of a concomitant change in sea water chemistry has been documented^19^.

How much time is represented by the hiatus and the microbial limestone was still unknown. A new thorough revision of the conodont biochronology by means of Unitary Association Zones (UAZs) highlighted that the conodont record around the PTB in shallow water sections is still insufficient for reliable age control^23^. Correlation by means of carbon isotope stratigraphy with the radio-isotopically well calibrated Global Stratotype Section and Point (GSSP) in Meishan^24^ is hindered by the extreme condensation of this section^25^. However, the carbonate carbon isotope record from Musandam^11^ allows positing that the peak segment of the negative carbon isotope excursion (CIE) straddling the PTB is erased by the hiatus in shallow water records of South China^19^. Hence, the respective timings of the unconformity and of the microbial limestone are highly relevant for constraining the underlying mechanisms during the extinction and its immediate aftermath.

Here, we propose a new, precise and accurate timescale for the regressive event that generated the hiatus, and for the overlying microbial bloom and its cessation, based on high-resolution U-Pb ages from 11 volcanic samples obtained from four shallow water boundary sections in the Nanpanjiang Basin (Fig. 1). The chronology can be directly compared to U-Pb ages from deep water records of juxtaposed troughs of the same basin^25^. Some of the underlying key mechanisms are then discussed in the light of this new time-space framework.

## High-precision U-Pb dates

The full data table and analytical details are given in the supplement. The detailed descriptions of the sampled sections are available in the supplement and in ref. 19, respectively. Th-corrected ^206^Pb/^238^U dates are presented as weighted mean ages of selected zircon populations and their associated ±2σ internal (analytical) uncertainties (±x) in Figs 2 and 3, and as single zircon grain ^206^Pb/^238^U age ranked distribution plots in Supplementary Fig. S1. Uncertainty of ^206^Pb/^238^U weighted mean ages in Supplementary Fig. S1 is reported as 2σ internal uncertainty (±x), 2σ external uncertainty including tracer calibration (±y), and 2σ external uncertainty including tracer calibration and ^238^U decay constant uncertainty (±z).

**Figure 1:**
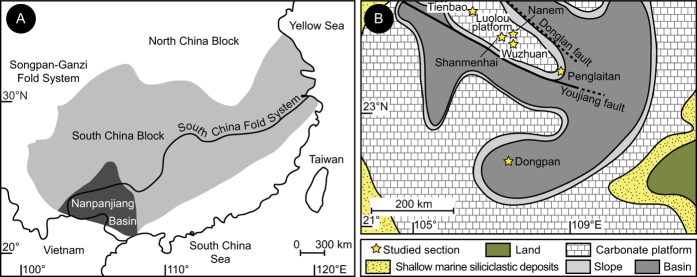
Tectonic map of South China and palaeogeographic map of the Nanpanjiang Basin. (**A**) Tectonic map of South China indicating the position of the North China Block, the South China Block, the Nanpanjiang Basin and the South China Fold System. (**B**) Late Permian to Early Triassic palaeogeographic map of the Nanpanjiang Basin showing the locations of the Shanmenhai, Nanem, Wuzhuan and Tienbao sections in the Luolou carbonate platform and of the deeper water Penglaitan and Dongpan sections. Both maps are created with Adobe Illustrator CS4 (https://helpx.adobe.com/creative-suite/kb/cs4-product-downloads.html).

**Figure 2:**
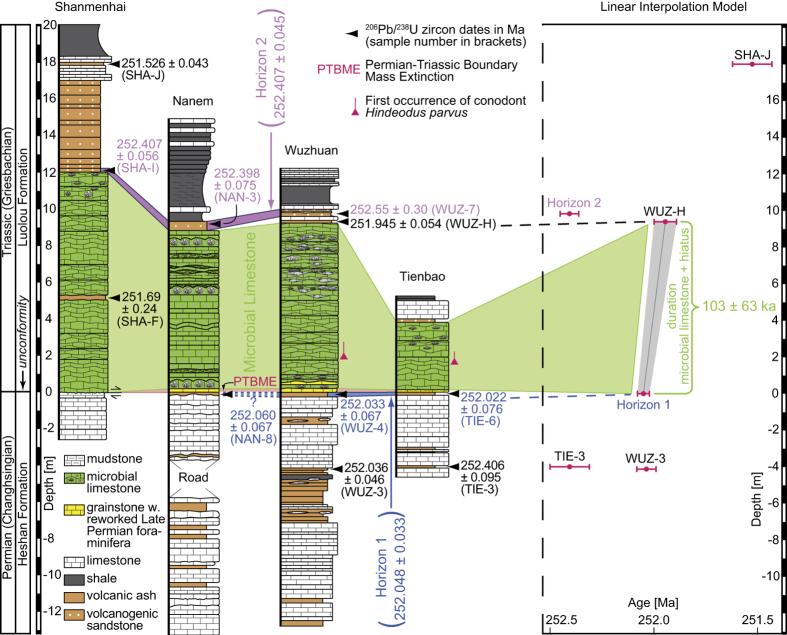
Stratigraphy and geochronology for the Shanmenhai, Nanem, Wuzhuan and Tienbao sections from late Changhsingian to Griesbachian showing ^206^Pb/^238^U weighted mean ages (in Ma; uncertainties are given by 2σ internal errors) of the investigated volcanic ashes and volcanogenic sandstone beds. Pooled ^206^Pb/^238^U weighted mean zircon population ages from ref. 26 for Horizon 1 (indicated in blue) and Horizon 2 (indicated in purple) are also given (see Supplementary Fig. S3). Though NAN-8 shows identical ^206^Pb/^238^U weighted mean age and similar zircon chemistry (see Supplementary Fig. S4a), the lack of apatite hampers definite correlation with Horizon 1. The first occurrences of the index conodont *Hindeodus parvus* are shown in their stratigraphic positions in Wuzhuan and Tienbao. The combined duration of the Triassic microbial limestone (indicated in green) and the underlying hiatus (marked by the arrows) of 103 ± 63 kyr is calculated by linear interpolation between Horizon 1 and WUZ-H, which bracket this period in Wuzhuan. The linear interpolation model is presented with its median (middle grey line) and its associated 95% confidence interval (grey area). In the linear interpolation model, red horizontal bars indicate ^206^Pb/^238^U weighted mean ages of dated volcanic levels in their stratigraphic position. MSWD = mean square of weighted deviates.

Five volcanic ash layers were sampled within the uppermost ~4 m of the Late Permian Heshan Fm. and six volcanic samples in the lowermost ~18 m of the Early Triassic Luolou Fm. (Fig. 2) in shallow water PTB sections from the Nanpanjiang Basin. Unlike in Shanmenhai where the PTB is obscured by low angle small scale faults, the topmost Permian bed immediately underlying the hiatus in Nanem (NAN-8), Wuzhuan (WUZ-4) and Tienbao (TIE-6) is represented by an ash layer (Fig. 2). This ash layer has been correlated in Wuzhuan, Tienbao and the deeper water section of Penglaitan by similar zircon and apatite chemistry and identical U-Pb zircon ages, suggesting origin from the same volcanic eruption (ref. 26 and see Supplementary Figs S3a and S4a). WUZ-4 and TIE-6 yield respective dates of 252.033 ± 0.067 Ma (N = 6; MSWD = 0.33) and 252.022 ± 0.076 Ma (N = 3; MSWD = 0.63), which overlap within internal errors. Based on this temporal coincidence and identical zircon and apatite chemistry, a pooled weighted mean age of 252.048 ± 0.033 Ma (N = 16; MSWD = 0.46), also including PEN-28, has thus been proposed for this last Permian marker bed in Wuzhuan, Tienbao and Penglaitan (referred to as Horizon 1^26^). Identical origin of NAN-8 due to similar zircon age (252.060 ± 0.067 Ma; N = 4; MSWD = 0.53) and chemistry is plausible (Supplementary Fig. S4a). It is also worth noting that WUZ-4 and TIE-6 are ca. 100 km far apart, which illustrates the lateral extension of this marker bed (Fig. 1).

The next underlying Permian ash sample occurs 4 m below the hiatus in Wuzhuan (WUZ-3) and represents the youngest layer of a 3 m thick succession of ash falls intercalated with subordinate limestone beds and lenses (Fig. 2). WUZ-3 yields a date of 252.036 ± 0.046 Ma (N = 6; MSWD = 0.05). The next underlying Permian ash sample in Tienbao (TIE-3) is also 4 m below the hiatus, but yields a date of 252.406 ± 0.095 Ma (N = 4; MSWD = 0.36). This indicates lower sediment accumulation rates for the Heshan Fm. at Tienbao, which is consistent with the modest (3.8 m) thickness of the microbial limestone at the base of the overlying Luolou Fm.

The lowermost Triassic ash layer occurs ~5 m above the base of the microbial limestone at Shanmenhai (SHA-F). SHA-F is not a typical air-fall tuff since its volcaniclastic material is diluted in a 0.1 m thick limestone bed intercalated in the microbial limestone. SHA-F yields a comparatively lower precision date of 251.69 ± 0.24 Ma (N = 3; MSWD = 0.99). The top of the microbial limestone is capped by an ash layer in Wuzhuan (WUZ-H) that is rarely preserved between the microbial limestone and an ubiquitous volcanogenic sandstone. WUZ-H provides an age of 251.945 ± 0.054 Ma (N = 8; MSWD = 0.54). At Shanmenhai, Nanem and Tienbao, this intervening ash layer is missing and the volcanogenic sandstone directly rests on top of the microbial limestone. Zircon and apatite chemistry were shown to have a uniform signature for all samples (SHA-I, NAN-3, WUZ-7) from this volcanogenic sandstone, which was labeled Horizon 2 (ref. 26; see Supplementary Fig. S3b). The re-deposited nature of Horizon 2 is confirmed by its ca. 400 ka too old U-Pb age, which violates the chronological succession imposed by air-fall tuffs. However, U-Pb ages of SHA-I (252.407 ± 0.056 Ma; N = 6; MSWD = 0.84), NAN-3 (252.398 ± 0.075 Ma; N = 5; MSWD = 0.53), and WUZ-7 (252.55 ± 0.30 Ma, youngest zircon date) all overlap within internal errors. Given the cogenetic nature of the volcanogenic beds, zircon U-Pb dates can be pooled, and a weighted mean age of 252.407 ± 0.045 Ma (N = 12; MSWD = 0.67) can be calculated for Horizon 2 (ref. 26; see Supplementary Fig. S3b).

The stratigraphically youngest ash bed (SHA-J) is ~6 m above the top of the microbial limestone and ~0.8 m above the top of the volcanogenic sandstone interval at Shanmenhai. It yields a date of 251.526 ± 0.043 Ma (N = 10; MSWD = 0.23). This age gives an upper limit for the deposition of the volcanogenic sandstone, which represents the main and abrupt drowning event that ended the deposition of the microbial limestone^19^.

## Duration of the hiatus and microbial bloom

**Figure 3:**
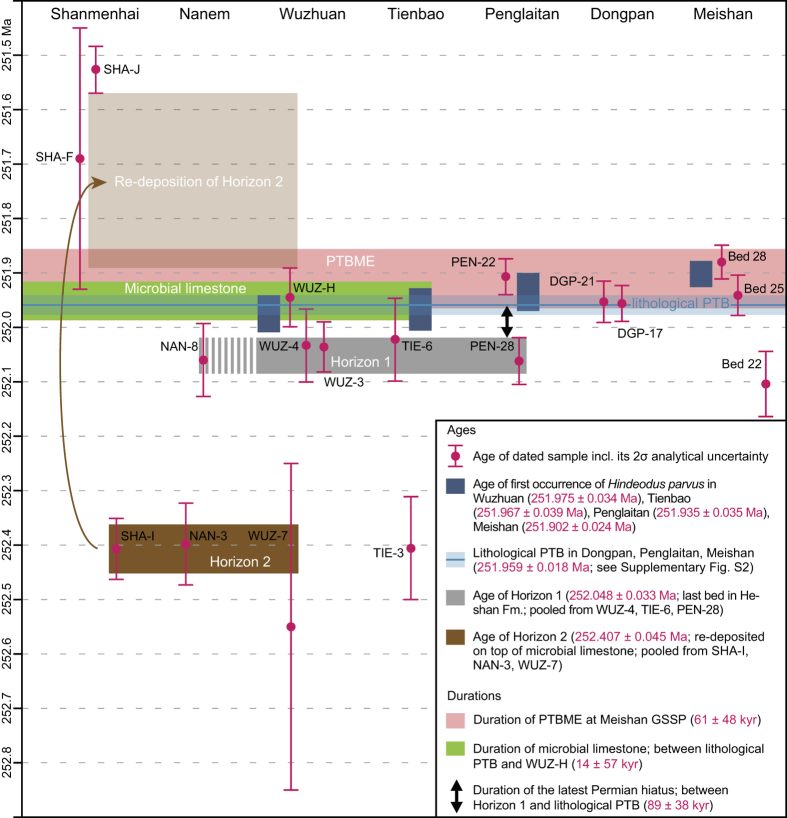
Comparison of ^206^Pb/^238^U weighted mean ages of volcanic ashes and volcanogenic sandstones from the shallow-marine Shanmenhai, Nanem, Wuzhuan and Tienbao sections with those from the deeper marine Dongpan and Penglaitan sections^25^ and the Meishan GSSP^24^. The ages of the first occurrence (FO) of *Hindeodus parvus* in Wuzhuan and Tienbao are from this study, the FOs of *H. parvus* in Penglaitan and Meishan are taken from ref. 25. The weighted mean age (see calculation in Supplementary Fig. S2) of the lithological Permian-Triassic boundary (PTB) in China is inferred from model ages of the boundary in Dongpan (bracketed by DGP-17 and DGP-21), in Meishan (bracketed by Bed 22 and Bed 25) and in Penglaitan (bracketed by PEN-28 and PEN-22)^25^. Ages of Horizon 1 and Horizon 2 are from ref. 26. The duration of the Permian-Triassic boundary mass extinction (PTBME; bracketed by Bed 25 and Bed 28) interval is taken from ref. 24. The respective durations of the hiatus and the microbial limestone are calculated from the time gap between Horizon 1 and the lithological PTB, and the time gap between the lithological PTB and WUZ-H. Uncertainty on durations is added in quadrature from 2σ analytical uncertainty on Horizon 1 (252.048 ± 0.033 Ma; N = 16; MSWD = 0.46), on the lithological PTB (251.959 ± 0.018 Ma; N = 3; MSWD = 2.2) and on WUZ-H (251.945 ± 0.054 Ma; N = 8; MSWD = 0.54). All associated uncertainties are given as 2σ analytical uncertainties.

Figure 3 compares the new chronological constraints from the shallow water sections of the Luolou Platform to those of the deeper water sections at Penglaitan and Dongpan^25^, and to the Meishan GSSP^24^. The lithological PTB is well constrained in terms of U-Pb dates at Dongpan, Penglaitan and Meishan^25^. The application of probabilistic age-depth models revealed the synchronicity of the formational PTBs in these sections, allowing to calculate a weighted mean age of 251.959 ± 0.018 Ma (N = 3; MSWD = 2.2; see Supplementary Fig. S2) for the PTB in China, as shown by the reference time line in Fig. 3. With the exception of its younger first occurrence (FO) in Meishan, all FOs of *Hindeodus parvus* intersect within errors with the age of the lithological PTB (Fig. 3). Furthermore, all FOs are either included or intersect with the extinction interval in Meishan, thus highlighting that the extinction interval and the system boundary cannot be resolved within the 40 ka uncertainty of the U-Pb dates. Hence, the end-Permian mass extinction (e.g., ref. 5) as previously defined and referred to in the literature is here more accurately named the Permian-Triassic *boundary* mass extinction (PTBME). The duration of the PTBME interval at Meishan has been estimated to 61 ± 48 kyr^24^.

A duration of 89 ± 38 kyr for the hiatus in shallow marine sections is derived from the late Permian Horizon 1 and the lithological PTB (Fig. 3). Based on the correlation of conodont UAZs, the inferred duration for the hiatus at the formational PTB in the Great Bank of Guizhou is 61 ± 48 kyr^23^, which is in agreement with our new estimate of 89 ± 38 kyr for the shallow water sections of the Luolou Platform. Another independent line of evidence is found in the carbon isotope records of Wuzhuan and Tienbao (Supplementary Fig. S5a). These indicate that a large segment of the negative CIE of the PTB in deeper water sections of Meishan and Dongpan is within the hiatus in Tienbao and Wuzhuan shallow water sections (Supplementary Fig. S5b). Subtracting the duration of 89 ± 38 kyr for the hiatus from the combined, linear interpolated duration of the hiatus and the microbial limestone of 103 ± 63 kyr (as inferred by linear interpolation between the pooled weighted mean ages of Horizon 1 and the weighted mean age of WUZ-H; Fig. 2), yields a duration of 14 ± 57 kyr for the microbial limestone alone (Fig. 3).

The microbial limestone shows striking lateral changes in thickness. It varies from 3.8 m at Tienbao to 9.1 m at Wuzhuan, although its duration may not depart significantly from 14 ± 57 kyr. Therefore, estimates of growth rate of the basal Triassic microbial limestone vary from >5.4 cm/ka at Tienbao to >13.0 cm/ka at Wuzhuan (Supplementary Fig. S5a). These estimates are all in agreement with the maximum growth rate of recent marine stromatolites (40 cm/ka; ref. 27) and with the duration of the microbial limestone in Wuzhuan recently proposed by ref. 21. The bathymetric differentiation of the microbial limestone is also corroborated by the carbonate carbon isotope records from Wuzhuan and Tienbao, whose basal Triassic end of the youngest negative shift is more expanded in Wuzhuan than in Tienbao (Supplementary Fig. S5a). Absence of the negative CIE in microbial limestone bearing sections^20^ supports the extension of the PTB hiatus into basal Griesbachian shallow water records (see Supplementary Fig. S5b). The presence of the widespread volcanogenic sandstone (Horizon 2) blanketing the microbial limestone also demonstrates that the cessation of this peculiar facies was nearly synchronous within the Luolou Platform. The pooled weighted mean age of Horizon 2 (252.407 ± 0.045 Ma) is also in agreement with the age of the volcanogenic sandstone DGP-18 from the deeper water section of Dongpan (252.56 ± 0.26 Ma; N = 1; youngest zircon date), where it occurs 0.5 m above the PTB (ref. 25 and see also Supplementary Fig. S4b). Additionally, DGP-18 shows identical zircon chemistry with Horizon 2, but apatite chemistry reveals a large spread of halogen and trace element composition. This spread either reflects different apatite composition than Horizon 2–thus precluding origin from a single source–or indicates alteration of the primary apatite chemical fingerprint. Assuming that the alteration hypothesis is correct, we infer that the first 0.5 m of basal Triassic black shales in Dongpan correlate with the first 9.5 m basal Triassic strata in Wuzhuan, which translates into a sediment accumulation rate of >0.6 cm/ka for the black shales at the base of the Ziyun Fm. in Dongpan (Supplementary Fig. S5b). An additional line of evidence in support of this inferred sediment accumulation rate of >0.6 cm/ka is found in the compatible >0.19 cm/ka sediment accumulation rate based on the age difference between the lithological PTB (251.959 ± 0.018 Ma) and the first basal Triassic ash bed DGP-21 (251.953 ± 0.038 Ma) in Dongpan^25^. In comparison to the >3.6 cm/ka obtained for the uppermost two meters of the Permian Dalong Fm. in Dongpan^25^, the minimum sediment accumulation rate of the basal Triassic black shales shows a six-fold decrease across the PTB. As there are no fundamental differences in bathymetry between the Dalong Fm. and the base of the Ziyun Fm., the strongly reduced sediment accumulation rate of the basal Triassic black shales of the Ziyun Fm. likely relates to a lower weathering rate, in agreement with the dry and cool Griesbachian climate drawn from the terrestrial plant record^28^. This climatic interpretation is also supported by clay mineralogy of the Shangsi section (northwestern South China Block) that revealed an accelerated aridification at the PTB^29^.

Since the time interval during which microbial limestone thrived on uplifted blocks only correlates with the basal 0.5 m of the >5 m black shale interval of adjacent downthrown blocks, the much later cessation of black shale deposition does not support common underlying paleoceanographic causes or relations for these two contrasted depositional environments (e.g., upwelling of nutrient-rich and alkaline waters^30^). Moreover, this correlation does not support a cessation of the microbial limestone caused by the disruption of a regionally stratified water column, or by any change in water chemistry common to both uplifted blocks and intervening troughs.

## Discussion

In the Nanpanjiang pull apart Basin, Late Permian to Early Triassic lateral changes in depositional setting and sediment accumulation rate were controlled by both eustatic sea-level changes and episodes of regional synsedimentary faulting^19^. Disentangling the effects of eustatic sea-level changes from those of regional tectonics is always delicate. However, dense U-Pb age control along with chemistry of accessory minerals allows recognition of synchronous tephrostratigraphic markers and the construction of a reliable absolute time framework^26^.

The new U-Pb dates presented here question the view that the main extinction event is older than the PTB (e.g., refs 31 and 32). Within our decamillennial age uncertainty, the main episode of the mass extinction, as recorded in the Meishan GSSP and elsewhere, can neither be distinguished from the revised paleontological definition of the PTB^23^, nor from formational boundaries associated with the PTB^25^. A converging conclusion was also reached by ref. 33. This revised timing has direct implications for the identification of the mechanistic causes of the PTBME.

The mainstream claim that the mass extinction occurred “during the transgressive pulse when anoxic bottom waters often became extensive”^4^ is untenable in the view of our timing from the South Chinese record with its locally restricted occurrences of Griesbachian anoxic marine deposits^15^,^20^,^34^,^35^,^36^. The duration of 89 ± 38 kyr for the hiatus in the Luolou Platform can only be explained by a short term marine regression of glacio-eustatic origin possibly combined with submarine dissolution of carbonate through acidification of surface waters. In the studied sections, both mechanical and chemical erosion can be called upon for the genesis of the hiatus. Mechanical erosion is supported by occasional deposition of high-energy grainstone, which exclusively contains reworked Permian faunas, within the base of the microbial limestone^19^. A new argument supporting shallow marine dissolution of the uppermost part of the Heshan Fm. is provided by the wide lateral extension of Horizon 1, the youngest preserved bed of the Permian Heshan Fm. In all sections where this horizon was recognized, it rests directly beneath the PTB hiatus without intervening Permian sedimentary rocks. Assuming that the obtained sediment accumulation rate of >6.6 cm/ka for the last preserved 4 m of the Heshan Fm. (Supplementary Fig. S5b) remained constant and considering the duration of the Permian part of the hiatus (89 ± 38 kyr), at least 3.4 m to 8.4 m of missing latest Permian rocks can be inferred. Generating the 89 ± 38 kyr gap - corresponding to a minimum of 3.4 m to 8.4 m of strata–with preservation of the same lower limit (i.e. Horizon 1) can hardly be explained by non-deposition or by mechanical erosion only. Ash layers such as Horizon 1 are extremely unlikely to resist subaerial and submarine mechanical erosion. The peculiar stratigraphic position of this bed as well as its remarkable lateral extension into the deeper water Penglaitan section^26^ some 300 km to the ESE from the Luolou Platform strongly suggests that it may have acted as a chemical shield against submarine dissolution caused by acidic waters. In the Luolou Platform, we obtain a duration of 14 ± 57 kyr for the deposition of the basal Triassic microbial limestone, whose growth and associated metazoan fauna excludes any coeval acidic and oxygen-deficient surface waters. The cessation of the microbial limestone in the Luolou Platform was caused by a sharp increase in base level that was manifested by a siliciclastic blanketing that deposited both volcanogenic sandstone (Horizon 2) and mudstone^19^. An episode of regional tectonic subsidence and the global sea-level rise may have jointly led to this cessation, but a concomitant change of water chemistry lacks any evidence^19^. Yet, the water depth, siliciclastic free and alkaline waters required for microbialite growth are in marked contrast with the earlier acidic and low stand conditions that prevailed during the 89 ± 38 kyr hiatus.

All these observations from the Nanpanjiang Basin as well as from other relevant sections world-wide must be integrated into a coherent causes and effects model. In this endeavor, the volcanogenic sulfur aerosol-driven model proposed by refs 37 and 38 provides the most parsimonious working frame, with the greatest explanatory power. Stratospheric injection of volcanogenic sulfur volatiles and subsequent condensation into aerosols are seen as the proximal cause for brief climate cooling^39^,^40^ and ensuing global regression^41^, provided that the cooling was profound enough to store water as terrestrial ice. Sulfur volatiles also provide a direct mechanism for ocean acidification. Abrupt cooling likely resulted from the atmospheric injection of both volcanogenic and remobilized SO_2_ and H_2_S from early Paleozoic evaporites by the initial emplacement of dykes and sills of the Siberian Traps^42^,^43^. This scenario simultaneously accounts for acidification of ocean surface waters and for the global regression. The synergistic effects of shrunken marine habitats on continental shelves through a global eustatic regression, of fast temperature drop down, and of substantial acidification are all compatible with the new timing proposed here. Moreover, paleontological evidence (e.g., ref. 44), facies interpretations and sediment accumulation rates are all compatible with the volcanogenic sulfur aerosol-driven model for the PTBME.

Subsequent and slower accumulation of CO_2_ derived from the basaltic effusions of the Siberian Traps and from burned Permian coal best account for successive warming and stepwise eustatic sea-level rise that first saw the deposition of the microbial limestone. The order of magnitude of the initial stage of the transgression in the Nanpanjiang Basin can be derived from the water depth (0 to 30 m) of present-day most common habitat of stromatolites^27^. Average Griesbachian pCO_2_ levels documented from plant cuticles^45^ may also have stimulated CaCO_3_ fixation via photosynthesis by cyanobacteria in equatorial surface waters, thus promoting the deposition of microbialites where normally oxygenated and clastic-free surface waters prevailed^19^. A subsequent and more substantial build up of pCO_2_ is needed to account for the following sea-level rise around the Griesbachian-Dienerian boundary^34^,^46^, for increased weathering rates^47^, for more frequent anoxia on continental shelves^34^,^48^, and for the largest ecological and concomitant change of terrestrial plants^30^. Our revised model is at striking variance with the long held mantra of an expanded oxygen minimum zone (e.g., ref. 6) as a leading kill mechanism for the PTBME. It must also be stressed that in our model large scale anoxia first comes into the play during Dienerian times. The “lethally” hot temperatures that were subsequently proposed as second kill mechanism^5^,^6^ are also irreconcilable with our model that involves a glacio-eustatic low-stand at the PTB. It is worth noting that these extremely hot temperatures are all derived from the South Chinese record^49^,^50^, whose equatorial position is the least climate sensitive configuration. It is also worth noting that all PTB sections^46^, from which temperature estimates were obtained, suffer from extreme condensation and hiatuses. Moreover, fractionation coefficients used for reconstructing sea-surface temperatures from oxygen isotopic values of biogenic phosphate^51^ rest on the multiple assumptions of an ice-free world, constant salinity and stable pH^49^, irrespective of the utilized analytical method (isotope ratio mass spectrometry or *in situ* secondary ion mass spectrometry). Yet, none of these prerequisites is valid within the frame of the volcanogenic sulfur aerosol-driven model. For all these reasons, available PTB temperature reconstructions based on oxygen isotopes from biogenic phosphate cannot be taken at face value. Abrupt drop down of sea surface temperature, shrunken habitats on shelves resulting from a global low stand, and short-term acidification may all have synergistically triggered the PTBME. In many aspects, the environmental upheavals linked with the PTBME (i.e. short-lived cooling followed by longer term warming) tend to parallel the most recent model proposed for the Triassic-Jurassic boundary mass extinction event^52^, thus leading to a more unifying view of causes and effects of large igneous provinces.

## Additional Information

**How to cite this article:** Baresel, B. *et al*. Timing of global regression and microbial bloom linked with the Permian-Triassic boundary mass extinction: implications for driving mechanisms. *Sci. Rep.* **7**, 43630; doi: 10.1038/srep43630 (2017).

**Publisher's note:** Springer Nature remains neutral with regard to jurisdictional claims in published maps and institutional affiliations.

## Supplementary Material

Supplementary Information
