# Supplementary material for: Timing of global regression and microbial bloom linked with the Permian-Triassic boundary mass extinction: implications for driving mechanisms
Source: Sci Rep. 2017 Mar 6;7:43630. doi: 10.1038/srep43630 (PMC5338007; doi:10.1038/srep43630)
Supplement: Supplementary Information [file srep43630-s1.pdf]

Timing of global regression and microbial bloom linked with the Permian-Triassic boundary mass extinction: implications for driving mechanisms

Björn Baresel [1*], Hugo Bucher [2], Borhan Bagherpour [2], Morgane Brosse [2], Kuang Guodun [3], Urs Schaltegger [1]

[1] Department of Earth Sciences, University of Geneva, Rue des Maraîchers 13, 1205 Geneva, Switzerland

[2] Paleontological Institute and Museum, University of Zurich, Karl Schmid-Strasse 4, 8006 Zurich, Switzerland

[3] Guangxi Bureau of Geology and Mineral Resources, Jiangzheng Road 1, 530023 Nanning, China

[*] corresponding author: bjorn.baresel@unige.ch

U-Pb geochronology of zircon

Sample preparation and U-Pb chemical abrasion isotope-dilution thermal ionization mass spectrometry (CA-ID-TIMS) analysis of zircon were carried out at the University of Geneva. The rock samples were crushed and milled, and the powders were wet-sieved to remove the clay fraction. Heavy minerals were isolated using methylene iodide. Populations of euhedral to subhedral zircon grains with maximum diameters ≤250 μm were microscopically inspected. No pre-imaging (such as cathodoluminescence or backscattered electron imaging) of the internal structure of zircon was conducted prior to analysis since it would result in significant loss of zircon volume and substantially increased uncertainty of the U-Pb zircon CA-ID-TIMS dates. However, the high age resolution of the U-Pb zircon CA-ID-TIMS method is sufficient enough to identify considerably older xeno- and ante-crystic zircons.

Euhedral crystals were picked for annealing at 900°C for ~48 h, followed by chemical abrasion with 40% HF and trace HNO_3 in pressurized dissolution 200 μl Ludwig-style capsules in a PARR vessel at 180°C for 18 h to minimize Pb loss effects (1). After several washing steps with water, 6 N HCl, and 3 N HNO_3, single crystals were loaded in the same 200 μl Ludwig-style capsules, spiked with ~4 mg of the EARTHTIME ^{202}Pb-^{205}Pb-^{233}U-^{235}U tracer solution (hereafter referred to as ET2535; 2) and dissolved in ~70 μl 40% HF and trace HNO_3 at 210°C for 48 h. After dissolution, samples were dried, dissolved again in 6 N HCl at 180°C for 12 h, dried down and dissolved again in 3 N HCl. U and Pb were collected in 3 ml Savillex beakers after separation in a modified single 50 μl column anion exchange chemistry (3) and dried down with a drop of 0.05 M H_3PO_4. They were loaded on a single outgassed Re filament with a Si-gel emitter modified from ref. 4. Measurements of U and Pb isotopes were performed on a Thermo TRITON thermal ionization mass spectrometer utilizing the ET2535 tracer calibration version

3.0 defined by ref. 2. Pb isotopes were measured in dynamic mode on a MasCom secondary electron multiplier with a deadtime of 23 ns. Instrumental mass fractionation was corrected using the fractionation factor derived from the measured $^{202}Pb/^{205}Pb$ ratio relative to a true value of 0.99924. $BaPO_2$ interferences on mass 202 to 205 were corrected by determining $^{138}Ba^{31}P^{16}O^{16}O$ concentration on mass 201 assuming natural abundance of ^{138}Ba of 71.7%. No correction was applied for isobaric interference of Tl on mass 205 (natural abundance of ^{205}Tl = 70.48% and ^{203}Tl = 29.52%) since routine check of Re filaments yielded negligible concentrations on mass 203. U isotopes were measured in static mode on Faraday cups equipped with 10^{12} Ω resistors as UO_2^+ and measured ratios were corrected for isobaric interferences of $^{233}U^{18}O^{16}O$ on $^{235}U^{16}O^{16}O$ using $^{18}O/^{16}O$ of 0.00205 ± 0.00004 (2σ), measured on large U500 loads, and for mass fractionation using the measured $^{233}U/^{235}U$ ratio relative to a value of 0.99506, assuming a sample $^{238}U/^{235}U$ ratio of 137.818 ± 0.045 (2σ; 5). Raw data were statistical filtered by using the Tripoli program, followed by data reduction including correct uncertainty propagation and online data visualization using U-Pb_Redux software (6,7). U-Pb ratios and dates were calculated relative to a tracer $^{235}U/^{205}Pb$ ratio of 100.23 ± 0.046% (2σ; 2). All common Pb in the analyses was assumed to be procedural blank yielding a long-term average $^{206}Pb/^{204}Pb$ of 18.469 ± 0.458, $^{207}Pb/^{204}Pb$ of 15.471 ± 0.320, $^{208}Pb/^{204}Pb$ of 38.011 ± 0.484 (uncertainties are given as 2σ) and an average of 0.44 pg during the course of this study. All uncertainties associated with weighted mean $^{206}Pb/^{238}U$ ages are at the 95% confidence level and reported as ±x, with x as analytical (internal) uncertainty. If the calculated dates are to be compared with other U-Pb laboratories not using the EARTHTIME tracer solution, ±y should be used which includes the systematic (external) uncertainty associated with the tracer calibration (0.03%). If dates are compared with other chronometers such as Ar-Ar, ±z should be used which also includes the ^{238}U decay

constant uncertainty (0.05%). All $^{206}Pb/^{238}U$ single-grain ages have been corrected for initial ^{230}Th-^{238}U disequilibrium assuming Th/U_{magma} of 3.00 ± 1.00 (2σ). This should best reflect the Th/U of the whole rock. Th-corrected $^{206}Pb/^{238}U$ dates are on average 80 kyr older than the equivalent uncorrected dates when applying this correction. The U-Pb isotopic results are presented as single-grain zircon $^{206}Pb/^{238}U$ age ranked distribution plots including their weighted mean $^{206}Pb/^{238}U$ zircon population ages with uncertainties reported as ±x/y/z in Fig. S1. The full data table is given in Tab. S1.

Sampling and U-Pb dates

U-Pb zircon chronology was carried out on single zircon crystals from volcanogenic sandstones and airfall volcanic ashes which are intercalated in shallow-marine sedimentary sequences of the Nanpanjiang Basin, south China. The study of such horizons is referred to as tephrochronology. A prior assumption in tephrochronology is that the age of zircon crystallization closely approximates that of the volcanic eruption and subsequent ash bed deposition (e.g., 8). Usually this assumption is valid, although zircon ages from tephra may be biased by time lags between crystallization and eruption or by Pb loss. This potential bias of zircon U-Pb dates was also considered to contribute to systematic offsets between the U-Pb and other radioisotopic systems such as ^{40}Ar-^{39}Ar (e.g., 9,10). The high temporal resolution of ID-TIMS geochronology often results in complex zircon age populations, reflecting prolonged zircon growth and magma residence, but on the other hand also allows to exclude considerably older xeno-, ante- or auto-crystic zircons which do not reflect the final crystallization stage in the last interstitial melt of the magmatic system. However, even with the use of the latest improvements of the U-Pb dating technique, such as the development of the chemical abrasion procedure (1), Pb loss phenomena in zircon have not yet been entirely erased. Hence, we assume that the youngest zircon population that yields a statistically robust weighted mean age (mean square of weighted deviates [MSWD] $\leq$ 1.00) should best reflect the age of the volcanic eruption and subsequent deposition of the ash bed.

Shanmenhai section

The Shanmenhai section is situated at 24°24'51.90"N and 107°2'27.50"E northwest of Bama in the province Guangxi, south China.

Sample SHA-F

Sample SHA-F was taken in the Triassic Luolou Fm. within the microbial limestone unit ~5 m above its base and represents a 10 cm thick volcanogenic bed. All five dated zircons are concordant within analytical error, but the youngest grain ($^{206}Pb/^{238}U$ age of 247.13 ± 0.15 Ma) shows unresolved lead loss and was discarded since it strongly violates the stratigraphic superposition with respect to all other dated volcanic beds. Subsequent, the three youngest grains define a cluster with a weighted mean $^{206}Pb/^{238}U$ age of 251.69 ± 0.24/0.25/0.36 Ma (MSWD = 0.99) for the deposition of SHA-F. Incorporation of the oldest zircon ($^{206}Pb/^{238}U$ age of 252.25 ± 0.24 Ma) into the mean age calculation would lead to a statistically flawed MSWD of 4.4. The major sources of analytical uncertainty (and their percentage contributions) are for the SHA-F.2 zircon analysis related to the correlated uncertainty of the U isotopes measurement ($^{265}UO_2/^{267}UO_2$, $^{270}UO_2/^{267}UO_2$; contribution of 97.9%) and to the correlated uncertainty of the Pb isotopes measurement ($^{204}Pb/^{205}Pb$, $^{206}Pb/^{205}Pb$, $^{208}Pb/^{205}Pb$; 1.5%). That the sources of analytical uncertainty can vary, is shown for the SHA-F.3 zircon analysis where the major sources are the uncertainty related to $^{206}Pb/^{204}Pb_{blank}$ composition (58.5%), followed by the correlated uncertainty of the U isotopes measurement ($^{265}UO_2/^{267}UO_2$, $^{270}UO_2/^{267}UO_2$; 26.9%) and the correlated uncertainty of the Pb isotopes measurement ($^{204}Pb/^{205}Pb$, $^{206}Pb/^{205}Pb$, $^{208}Pb/^{205}Pb$; 11.3%).

Sample SHA-I

The overlying sample SHA-I was taken in the Triassic Luolou Fm. ca. 12 m above the base of the microbial limestone and indicates the first bed of a 5 m thick volcanogenic sandstone interval, which is situated directly on top of the microbial limestone in Shanmenhai. Ten zircon crystals were analyzed, resulting in scattered $^{206}Pb/^{238}U$ dates of 252.34 ± 0.12 Ma to 253.59 ±

0.36 Ma. The six youngest zircons yield a weighted mean $^{206}Pb/^{238}U$ age of 252.407 ± 0.056/0.086/0.28 Ma (MSWD = 0.84) for the deposition of SHA-I. Incorporation of the slightly older SHA-I.13 zircon grain ($^{206}Pb/^{238}U$ age of 252.536 ± 0.075 Ma) into the mean age calculation would lead to a statistically flawed MSWD of 2.0. Zircon dates from this volcanic bed spread over more than 1 Myr. This indicates recycling of older volcanic material via sedimentary or magmatic processes since the magmatic residence time of autocrystic zircon is usually up to 0.4 Myr (depending on the size of the magmatic system and the magma production rate; 11).

Sample SHA-J

The stratigraphically youngest ash layer SHA-J represents a 5 cm thick, fine grained bed and occurs 18 m above the base of the microbial limestone. Analyses of ten individual zircon crystals yield a statistically significant cluster with a weighted mean $^{206}Pb/^{238}U$ age of 251.526 ± 0.043/0.078/0.28 Ma (MSWD = 0.23) representing the youngest zircon population of this ash bed. If only the youngest zircon ($^{206}Pb/^{238}U$ age of 251.48 ± 0.17/0.18/0.33 Ma) or the youngest zircon population consisting of the two youngest ($^{206}Pb/^{238}U$ age of 251.491 ± 0.072/0.098/0.29 Ma; MSWD = 0.04) or the three youngest ($^{206}Pb/^{238}U$ age of 251.496 ± 0.064/0.092/0.28 Ma; MSWD = 0.06) zircon grains would be selected, the deposition age of this ash bed would persist, but its precision would be substantially reduced. The major sources of analytical uncertainty (their percentage contribution is shown here for the representative SHA-J.1 zircon analysis) are in descending order related to the $^{206}Pb/^{204}Pb_{blank}$ composition (contribution of 45.9% to the internal uncertainty), to the U isotopes measurement ($^{265}UO_2/^{267}UO_2$, $^{270}UO_2/^{267}UO_2$; 32.8%), to the Pb isotopes measurement ($^{204}Pb/^{205}Pb$, $^{206}Pb/^{205}Pb$, $^{208}Pb/^{205}Pb$; 15.8%), to the Pb isotopes

measurement of the ET2535 tracer solution ($^{202}Pb/^{205}Pb$; 2.9%) and to the U_{blank} mass determination (2.1%).

Nanem section

The Nanem section is exposed at 24°24'3.70"N and 107°19'29.20"E at a roadcut north of Bama in the province Guangxi, south China.

Sample NAN-8

The ash layer NAN-8 was sampled in the Late Permian Heshan Fm. and occurs right below the hiatus. This 5-10 cm thick, argillaceous ash bed represents the last Permian bed in Nanem. Nine zircon crystals were analyzed, resulting in scattered $^{206}Pb/^{238}U$ dates of 251.94 ± 0.72 Ma to 253.93 ± 0.12 Ma. The four youngest zircons yield a weighted mean $^{206}Pb/^{238}U$ age of 252.060 ± 0.067/0.094/0.29 Ma (MSWD = 0.53) for the deposition of this ash bed. Since zircon dates from this ash bed spread over 2 Myr, they indicate incorporation of older volcanic material via sedimentary or magmatic recycling. The major sources of analytical uncertainty of this zircon dates are the U and Pb isotopes measurements.

Sample NAN-3

NAN-3 is situated 8.8 m above the base of the microbial limestone interval in Nanem and represents the base of a ca. 40 cm thick volcanogenic sandstone right on top of the microbial limestone. Nine zircon crystals were analyzed, resulting in scattered $^{206}Pb/^{238}U$ dates of 252.22 ± 0.51 Ma to 253.28 ± 0.15 Ma, where the five youngest zircons yield a statistically significant cluster with a weighted mean $^{206}Pb/^{238}U$ age of 252.398 ± 0.075/0.10/0.29 Ma (MSWD = 0.53). Incorporation of the slightly older zircon grains NAN-3.5 ($^{206}Pb/^{238}U$ age of 252.56 ± 0.14 Ma) and NAN-3.11 ($^{206}Pb/^{238}U$ age of 252.58 ± 0.13 Ma) into the mean age calculation would lead to

a statistically flawed MSWD of 1.8. The dominant source of analytical uncertainty for all NAN-3 zircon dates is the U isotopes analysis.

Wuzhuan section

The Wuzhuan section outcrops at 24°21'44.6" N and 107°20'02.00" E at a roadcut north of Bama in the province Guangxi, south China. Four volcanogenic samples were taken, two in the Permian Heshan Fm. and two in the Triassic Luolou Fm.

Sample WUZ-3

The stratigraphically oldest sample WUZ-3 occurs 4 m below the hiatus in Wuzhuan and represents the youngest layer of a 3 m thick succession of ash falls intercalated with subordinate limestone beds and lenses. Seven zircon crystals were analyzed, where the six youngest zircons yield a statistically significant cluster with a weighted mean $^{206}Pb/^{238}U$ age of 252.036 ± 0.046/0.080/0.28 Ma (MSWD = 0.05) for the deposition of ash bed WUZ-3. One zircon grain is remarkably older with a $^{206}Pb/^{238}U$ age of 253.41 ± 0.29 Ma. This might be an analytical issue since the dominant source of analytical uncertainty for this zircon date is the Pb isotopes measurement which mainly affects the accuracy of the zircon date, whereas all other zircon dates from WUZ-3 are dominated by the uncertainty of the U isotopes measurement, mainly affecting the precision of the dates.

Sample WUZ-4

WUZ-4 is a 15 cm thick ash bed directly underlying the hiatus. Ten zircon crystals were analyzed, resulting in scattered $^{206}Pb/^{238}U$ dates of 251.98 ± 0.18 Ma to 254.21 ± 0.19 Ma, where the six youngest zircons yield a statistically significant cluster with a weighted mean $^{206}Pb/^{238}U$ age of 252.033 ± 0.067/0.094/0.29 Ma (MSWD = 0.33) for the deposition of ash bed WUZ-4.

Incorporation of the slightly older zircon grain WUZ-4.1 ($^{206}Pb/^{238}U$ age of 252.45 ± 0.11 Ma) into the weighted mean age calculation would lead to a statistically flawed MSWD of 7.7 and would further violate the stratigraphic superposition with respect to the WUZ-3 ash bed below and the WUZ-H ash bed above. The dominant source of analytical uncertainty for all WUZ-4 zircon dates is the U isotopes measurement.

Sample WUZ-H

WUZ-H represents a 10 cm thick ash layer that rests directly on top of the microbial limestone in the Triassic Luolou Fm. of Wuzhuan. Eleven zircon crystals were analyzed, resulting in scattered $^{206}Pb/^{238}U$ dates of 251.83 ± 0.21 Ma to 253.06 ± 0.19 Ma, where the eight youngest zircons yield a statistically significant cluster with a weighted mean $^{206}Pb/^{238}U$ age of 251.945 ± 0.054/0.085/0.28 Ma (MSWD = 0.54) for the deposition of WUZ-H. Incorporation of the three older zircon grains into the weighted mean age calculation would lead to statistically flawed MSWDs of 3.4 to 18. The dominant sources of analytical uncertainty for all WUZ-H zircon dates are the U and Pb isotopes measurements and to some minor extent the $^{206}Pb/^{204}Pb_{blank}$ composition.

Sample WUZ-7

WUZ-7 represents a 10 cm thick volcanogenic sandstone stratigraphically above WUZ-H and is situated 9.5 m above the base of the microbial limestone unit. Nine zircon crystals were analyzed, resulting in scattered $^{206}Pb/^{238}U$ dates of 252.55 ± 0.30 Ma to 254.45 ± 0.62 Ma. Since the youngest zircon grain neither shows analytical irregularities, nor violates the stratigraphic superposition, we assume that its $^{206}Pb/^{238}U$ age of 252.55 ± 0.30/0.31/0.41 Ma (MSWD = 0.54 best reflects the eruption age of WUZ-7. Zircon dates from this volcanic bed spread over 2 Myr, which suggests incorporation of older volcanic material via sedimentary or magmatic recycling.

Incorporation of the older zircon grains into the weighted mean age calculation would lead to statistically flawed MSWDs of 3.0 to 8.4. The dominant sources of analytical uncertainty for the WUZ-7 zircon dates are the U and Pb isotopes measurements, except for WUZ-7.4 analysis, which is dominated by the uncertainty related to the $^{206}Pb/^{204}Pb_{blank}$ composition.

Tienbao section

The Tienbao section is located at 24°50'3.40"N and 106°29'20.90"E northwest of Leye in the province Guangxi, south China.

Sample TIE-3

TIE-3 represents a 10 cm thick, fine-grained ash bed and is situated 4.1 m below the hiatus in Nanem. Seven zircon crystals were analyzed, resulting in scattered $^{206}Pb/^{238}U$ dates of 252.30 ± 0.23 Ma to 255.73 ± 0.30 Ma, where the four youngest zircons yield a statistically significant cluster with a weighted mean $^{206}Pb/^{238}U$ age of 252.406 ± 0.095/0.11/0.29 Ma (MSWD = 0.36) for the deposition of TIE-3. Incorporation of the three older zircon grains into the weighted mean age calculation would lead to a statistically flawed MSWD of 30 to 91. The dominant sources of analytical uncertainty for the TIE-3 zircon dates are the U and Pb isotopes measurements, except for TIE-3.2 and TIE-3.5 which are dominated by the uncertainty related to the $^{206}Pb/^{204}Pb_{blank}$ composition.

Sample TIE-6

The ash bed TIE-6 was sampled in the Permian Heshan Fm. and represents a 10 cm thick, fine-grained ash bed, which is situated directly below the hiatus. Eight zircon crystals were analyzed, resulting in scattered $^{206}Pb/^{238}U$ dates of 251.92 ± 0.30 Ma to 253.94 ± 0.31 Ma, where the three youngest zircons yield a statistically significant cluster with a weighted mean $^{206}Pb/^{238}U$ age of

252.022 ± 0.076/0.10/0.29 Ma (MSWD = 0.63) for the deposition of TIE-6. Incorporation of the five older zircon grains into the weighted mean age calculation would lead to statistically flawed MSWDs of 7.3 to 80. The dominant sources of analytical uncertainty for the TIE-6 zircon dates are the U and Pb isotopes measurements and to some minor extent the $^{206}Pb/^{204}Pb_{blank}$ composition.

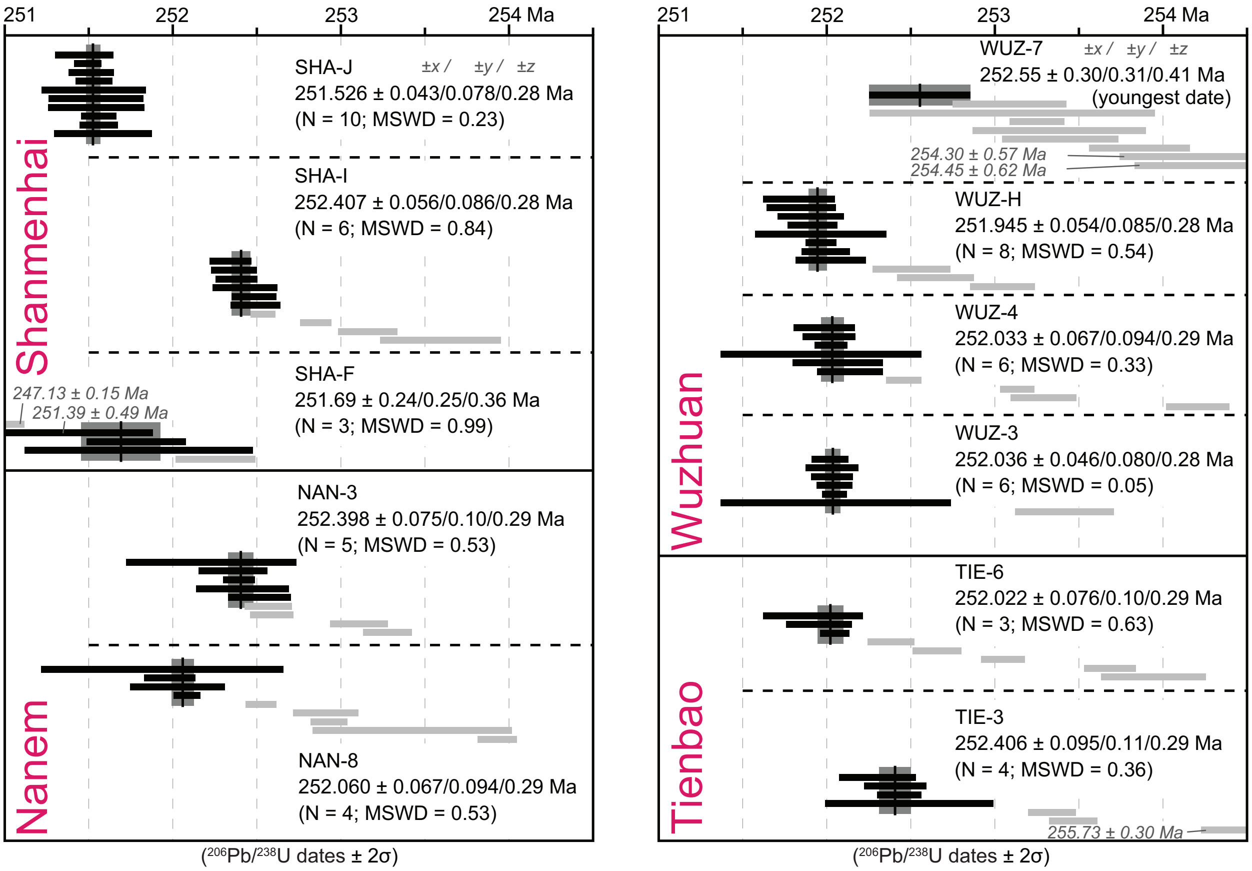

Figure S1. $^{206}Pb/^{238}U$ single-grain zircon analyses and weighted mean ages for Shanmenhai, Nanem, Wuzhuan and Tienbao volcanic ashes and volcanogenic sandstones. Each horizontal bar represents a single-grain zircon analysis including its 2σ analytical (internal) uncertainty whereas grey bars are not included in the weighted mean age calculation. Vertical lines represent the weighted mean age with the associated 2σ uncertainty (in grey). Uncertainty of the weighted mean age is reported as 2σ internal (±x), 2σ external uncertainty including tracer calibration (±y),

and 2σ external uncertainty including tracer calibration and ^{238}U decay constant uncertainty (±z); MSWD = mean square of weighted deviates.

Table S1. U-Pb single-grain zircon dates and isotopic data.

Fraction and sample	Dates (Ma)					Composition			Isotopic Ratios						
	206Pb/238U *a °°	±2σ (absolute)	207Pb/235U *a	±2σ (absolute)	Disc. (%) *b	Th/U *c	Pb (pg) *d	Pbc (pg) *e	206Pb/204Pb *f	206Pb/238U *g	±2σ (%)	207Pb/235U *g	±2σ (%)	207Pb/206Pb *g	±2σ (%)
Wuzhuan-H															
WUZ-H.1	252.025	0.204	252.022	0.825	0.33	0.62	22.52	0.66	2032	0.0399	0.08	0.2817	0.37	0.0513	0.35
WUZ-H.2	251.965	0.388	251.688	0.696	-0.81	0.72	11.46	0.15	4503	0.0398	0.16	0.2813	0.31	0.0512	0.25
WUZ-H.3	253.057	0.188	252.381	0.835	-2.48	0.71	8.27	0.16	2939	0.0400	0.08	0.2822	0.37	0.0512	0.35
WUZ-H.4	251.994	0.138	251.688	0.255	-0.93	0.67	81.08	0.29	16007	0.0399	0.06	0.2813	0.11	0.0512	0.08
WUZ-H.6	252.655	0.224	252.576	0.458	0.00	0.74	16.30	0.15	6295	0.0400	0.09	0.2824	0.20	0.0513	0.20
WUZ-H.7	251.847	0.201	251.419	0.909	-1.45	0.69	10.81	0.26	2443	0.0398	0.08	0.2810	0.41	0.0512	0.37
WUZ-H.8	252.510	0.227	252.326	0.905	-0.42	0.65	9.92	0.21	2776	0.0399	0.09	0.2821	0.41	0.0513	0.33
WUZ-H.9	251.916	0.144	250.864	0.910	-4.15	0.71	7.28	0.18	2283	0.0398	0.06	0.2803	0.41	0.0510	0.38
WUZ-H.10	251.833	0.210	251.612	0.486	-0.57	0.66	16.50	0.15	6336	0.0398	0.08	0.2812	0.22	0.0512	0.21
WUZ-H.12	252.097	0.203	250.544	0.948	-6.43	0.66	7.70	0.19	2338	0.0399	0.08	0.2799	0.43	0.0509	0.37
WUZ-H.13	251.966	0.086	251.386	0.603	-2.09	0.69	13.09	0.23	3343	0.0398	0.03	0.2809	0.27	0.0512	0.26
Wuzhuan-7															
WUZ-7.2	253.252	0.162	252.886	0.361	-1.17	0.66	18.77	0.19	5889	0.0401	0.07	0.2828	0.16	0.0512	0.14
WUZ-7.3	253.382	0.516	252.327	0.874	-4.11	0.64	12.83	0.25	2959	0.0401	0.21	0.2821	0.39	0.0511	0.32
WUZ-7.4	254.305	0.566	255.638	5.070	5.37	0.63	19.35	4.04	299	0.0402	0.23	0.2863	2.24	0.0516	2.24
WUZ-7.5	253.861	0.299	253.731	0.494	-0.19	0.64	20.94	0.27	4572	0.0402	0.12	0.2839	0.22	0.0513	0.19
WUZ-7.6	253.384	0.345	253.234	0.498	-0.27	0.63	25.14	0.25	6031	0.0401	0.14	0.2833	0.22	0.0513	0.16
WUZ-7.7	252.554	0.301	252.375	0.585	-0.40	0.68	16.73	0.17	5711	0.0399	0.12	0.2822	0.26	0.0513	0.21
WUZ-7.8	253.099	0.849	252.577	1.004	-1.82	0.64	18.35	0.34	3190	0.0400	0.34	0.2824	0.45	0.0512	0.26
WUZ-7.9	254.452	0.622	252.894	0.958	-6.32	0.62	15.86	0.23	4000	0.0402	0.25	0.2828	0.43	0.0510	0.33
WUZ-7.10	253.086	0.338	252.495	0.525	-2.11	0.62	23.37	0.19	7332	0.0400	0.14	0.2823	0.23	0.0512	0.16
Wuzhuan-4															
WUZ-4.1	252.455	0.107	252.435	0.857	0.22	0.92	13.32	0.40	1827	0.0399	0.04	0.2822	0.38	0.0513	0.37
WUZ-4.2	251.985	0.183	252.096	1.614	0.79	0.62	29.74	1.93	925	0.0398	0.07	0.2818	0.72	0.0513	0.72
WUZ-4.4	252.066	0.269	252.046	1.366	0.26	0.62	8.28	0.39	1278	0.0399	0.11	0.2818	0.61	0.0513	0.58
WUZ-4.5	252.013	0.157	251.899	0.720	-0.11	0.52	20.73	0.45	2791	0.0399	0.06	0.2816	0.32	0.0513	0.29
WUZ-4.6	252.025	0.657	252.115	0.704	0.68	0.80	54.63	0.43	7218	0.0399	0.27	0.2818	0.32	0.0513	0.17
WUZ-4.7	252.024	0.096	251.949	0.521	0.03	0.64	23.62	0.37	3720	0.0399	0.04	0.2816	0.23	0.0513	0.21
WUZ-4.8	253.288	0.194	253.084	1.741	-0.50	0.70	6.79	0.45	892	0.0401	0.08	0.2831	0.78	0.0513	0.77
WUZ-4.9	254.205	0.189	254.031	0.507	-0.40	0.86	12.19	0.14	4771	0.0402	0.08	0.2843	0.23	0.0513	0.19
WUZ-4.10	252.140	0.195	252.226	0.414	0.67	0.71	15.85	0.17	5485	0.0399	0.08	0.2820	0.19	0.0513	0.15
WUZ-4.11	253.131	0.101	253.197	0.667	0.57	0.82	7.43	0.16	2696	0.0400	0.04	0.2832	0.30	0.0513	0.28
Wuzhuan-3															
WUZ-3.3	252.028	0.156	251.847	0.820	-0.41	0.67	16.65	0.43	2288	0.0399	0.06	0.2815	0.37	0.0512	0.37
WUZ-3.4	252.055	0.680	252.210	2.035	0.93	0.88	24.21	0.42	3171	0.0399	0.28	0.2820	0.91	0.0513	0.75
WUZ-3.5	252.045	0.072	252.000	0.338	0.16	0.62	39.07	0.44	5242	0.0399	0.03	0.2817	0.15	0.0513	0.14
WUZ-3.6	252.029	0.123	252.066	1.073	0.47	0.78	11.81	0.44	1548	0.0399	0.05	0.2818	0.48	0.0513	0.47
WUZ-3.7	253.412	0.294	252.883	1.077	-1.83	0.52	9.17	0.19	2872	0.0401	0.12	0.2828	0.48	0.0512	0.39
WUZ-3.8	252.045	0.106	252.092	0.481	0.52	0.67	14.91	0.18	4852	0.0399	0.04	0.2818	0.22	0.0513	0.20

Table S1. U-Pb single-grain zircon dates and isotopic data.

Fraction and sample	Dates (Ma) 206Pb/238U *a °°	±2σ (absolute)	207Pb/235U *a	±2σ (absolute)	Disc. (%) *b	Composition Th/U *c	Pb (pg) *d	Pbc (pg) *e	Isotopic Ratios 206Pb/204Pb *f	206Pb/238U *g	±2σ (%)	207Pb/235U *g	±2σ (%)	207Pb/206Pb *g	±2σ (%)
WUZ-3.9	252.017	0.109	251.867	0.456	-0.28	0.64	16.32	0.17	5794	0.0399	0.04	0.2815	0.20	0.0513	0.20
Tienbao-6															
TIE-6.1	251.916	0.297	251.953	0.352	0.48	0.67	48.95	0.35	8239	0.0398	0.12	0.2816	0.16	0.0513	0.14
TIE-6.2	251.954	0.194	252.237	0.988	1.48	0.57	15.35	0.56	1649	0.0398	0.08	0.2820	0.44	0.0514	0.41
TIE-6.3	253.049	0.129	259.628	0.526	21.00	0.59	18.46	0.31	3486	0.0400	0.05	0.2914	0.23	0.0528	0.21
TIE-6.4	253.679	0.153	261.145	0.945	23.03	0.60	12.22	0.36	2017	0.0401	0.06	0.2933	0.41	0.0530	0.38
TIE-6.5	252.655	0.146	252.944	1.062	1.49	0.64	12.90	0.47	1607	0.0400	0.06	0.2829	0.47	0.0514	0.46
TIE-6.6	252.044	0.085	252.006	0.521	0.16	0.82	16.26	0.22	4224	0.0399	0.03	0.2817	0.23	0.0513	0.22
TIE-6.7	252.381	0.139	252.248	0.795	-0.20	0.61	9.50	0.22	2612	0.0399	0.06	0.2820	0.36	0.0513	0.34
TIE-6.8	253.942	0.314	254.012	2.145	0.62	0.56	3.26	0.22	906	0.0402	0.13	0.2842	0.95	0.0513	0.87
Tienbao-3															
TIE-3.2	252.490	0.500	259.566	5.958	22.25	0.82	13.97	3.33	253	0.0399	0.20	0.2913	2.60	0.0529	2.61
TIE-3.3	253.462	0.140	262.833	0.671	27.18	0.71	27.80	0.61	2648	0.0401	0.06	0.2954	0.29	0.0535	0.27
TIE-3.4	253.332	0.142	254.782	0.887	5.81	0.72	9.94	0.26	2242	0.0401	0.06	0.2852	0.39	0.0516	0.36
TIE-3.5	255.727	0.303	269.821	2.955	35.21	0.95	5.96	0.63	529	0.0405	0.12	0.3044	1.25	0.0546	1.23
TIE-3.6	252.413	0.188	252.484	1.703	0.61	0.73	13.49	0.85	929	0.0399	0.08	0.2823	0.76	0.0513	0.74
TIE-3.7	252.430	0.129	252.221	0.792	-0.53	0.70	11.23	0.31	2088	0.0399	0.05	0.2820	0.35	0.0512	0.36
TIE-3.8	252.302	0.228	252.351	1.580	0.54	0.65	8.24	0.50	986	0.0399	0.09	0.2821	0.71	0.0513	0.69
Shanmenhai-J															
SHA-J.1	251.560	0.101	251.411	0.897	-0.26	0.55	14.07	0.51	1673	0.0398	0.04	0.2810	0.40	0.0512	0.40
SHA-J.2	251.476	0.173	251.669	1.820	1.10	0.74	8.11	0.40	1179	0.0398	0.07	0.2813	0.82	0.0513	0.83
SHA-J.3	251.544	0.287	251.487	3.004	0.12	0.57	4.97	0.60	508	0.0398	0.12	0.2810	1.35	0.0513	1.34
SHA-J.4	251.543	0.280	251.238	3.035	-0.91	0.57	5.30	0.64	513	0.0398	0.11	0.2807	1.36	0.0512	1.35
SHA-J.5	251.561	0.114	251.494	0.679	0.07	0.61	8.16	0.16	2981	0.0398	0.05	0.2811	0.30	0.0513	0.28
SHA-J.6	251.531	0.109	251.780	0.835	1.36	0.48	8.11	0.18	2732	0.0398	0.04	0.2814	0.37	0.0513	0.36
SHA-J.8	251.494	0.080	251.490	0.620	0.34	0.51	7.20	0.15	2917	0.0398	0.03	0.2810	0.28	0.0513	0.29
SHA-J.9	251.587	0.288	251.761	2.041	1.02	0.80	3.26	0.15	1226	0.0398	0.12	0.2814	0.92	0.0513	0.84
SHA-J.10	251.513	0.133	251.267	0.951	-0.68	0.65	5.86	0.15	2222	0.0398	0.05	0.2808	0.43	0.0512	0.44
SHA-J.11	251.532	0.310	247.526	2.876	-19.43	0.48	1.74	0.17	661	0.0398	0.13	0.2761	1.31	0.0504	1.30
Shanmenhai-I															
SHA-I.3	252.429	0.194	252.467	0.936	0.48	0.70	13.62	0.45	1775	0.0399	0.08	0.2823	0.42	0.0513	0.40
SHA-I.4	252.342	0.123	252.262	0.697	0.00	0.69	19.17	0.46	2436	0.0399	0.05	0.2820	0.31	0.0513	0.30
SHA-I.6	253.160	0.172	253.195	0.824	0.46	0.72	19.16	0.49	2267	0.0400	0.07	0.2832	0.37	0.0513	0.35
SHA-I.7	252.380	0.123	252.326	0.389	0.12	0.61	39.98	0.47	5062	0.0399	0.05	0.2821	0.17	0.0513	0.15
SHA-I.8	252.489	0.147	252.500	0.623	0.37	0.67	31.62	0.70	2641	0.0399	0.06	0.2823	0.28	0.0513	0.26
SHA-I.9	252.481	0.135	252.416	0.951	0.06	0.73	19.04	0.60	1839	0.0399	0.05	0.2822	0.43	0.0513	0.41
SHA-I.10	252.848	0.090	252.817	0.539	0.20	0.70	26.15	0.46	3300	0.0400	0.04	0.2827	0.24	0.0513	0.23
SHA-I.13	252.536	0.075	252.625	0.481	0.68	0.77	28.23	0.46	3524	0.0399	0.03	0.2825	0.21	0.0513	0.21
SHA-I.14	253.587	0.360	253.519	0.985	0.06	0.67	15.62	0.45	2034	0.0401	0.14	0.2836	0.44	0.0513	0.39
SHA-I.15	252.363	0.136	252.486	0.398	0.83	0.67	36.42	0.48	4436	0.0399	0.05	0.2823	0.18	0.0513	0.16

Table S1. U-Pb single-grain zircon dates and isotopic data.

Fraction and sample	Dates (Ma) 206Pb/238U *a °°	±2σ (absolute)	207Pb/235U *a	±2σ (absolute)	Disc. (%) *b	Composition Th/U *c	Pb (pg) *d	Pbc (pg) *e	Isotopic Ratios 206Pb/204Pb *f	206Pb/238U *g	±2σ (%)	207Pb/235U *g	±2σ (%)	207Pb/206Pb *g	±2σ (%)
Shanmenhai-F															
SHA-F.2	251.389	0.493	251.437	0.619	0.52	0.72	45.27	0.65	4008	0.0398	0.20	0.2810	0.28	0.0513	0.18
SHA-F.3	252.253	0.238	252.282	2.384	0.44	0.73	5.52	0.51	642	0.0399	0.10	0.2820	1.07	0.0513	1.06
SHA-F.7	251.800	0.680	251.841	4.130	0.50	0.65	5.76	0.50	688	0.0398	0.28	0.2815	1.85	0.0513	1.70
SHA-F.8	247.127	0.154	247.045	0.316	-0.01	0.74	25.51	0.18	8091	0.0391	0.06	0.2755	0.14	0.0512	0.13
SHA-F.9	251.782	0.294	251.756	0.890	0.24	0.56	5.59	0.15	2254	0.0398	0.12	0.2814	0.40	0.0513	0.39
Nanem-3															
NAN-3.3	252.406	0.279	251.361	0.562	-4.10	0.67	15.89	0.21	4381	0.0399	0.11	0.2809	0.25	0.0511	0.20
NAN-3.4	253.104	0.171	252.419	0.422	-2.52	0.68	16.44	0.18	5349	0.0400	0.07	0.2822	0.19	0.0512	0.16
NAN-3.5	252.563	0.143	252.159	0.416	-1.33	0.64	26.11	0.31	4950	0.0399	0.06	0.2819	0.19	0.0512	0.17
NAN-3.6	252.507	0.187	252.878	0.563	1.81	0.65	27.00	0.47	3354	0.0399	0.08	0.2828	0.25	0.0514	0.22
NAN-3.7	253.276	0.146	252.785	0.446	-1.70	0.71	24.31	0.29	4909	0.0401	0.06	0.2827	0.20	0.0512	0.17
NAN-3.8	252.386	0.095	252.135	0.289	-0.70	0.67	20.02	0.16	7496	0.0399	0.04	0.2819	0.13	0.0512	0.12
NAN-3.10	252.350	0.207	252.067	0.472	-0.83	0.70	22.91	0.32	4170	0.0399	0.08	0.2818	0.21	0.0512	0.18
NAN-3.11	252.581	0.126	252.631	0.793	0.53	0.66	31.29	0.97	1886	0.0399	0.05	0.2825	0.35	0.0513	0.35
NAN-3.12	252.220	0.507	252.329	0.760	0.77	0.68	34.28	0.57	3521	0.0399	0.21	0.2821	0.34	0.0513	0.26
Nanem-8															
NAN-8.1	253.932	0.116	259.598	0.559	18.59	0.69	29.11	0.43	3907	0.0402	0.05	0.2913	0.24	0.0526	0.22
NAN-8.3	252.523	0.092	257.506	0.283	16.93	0.63	58.48	0.42	8123	0.0399	0.04	0.2887	0.12	0.0524	0.11
NAN-8.5	251.938	0.722	255.637	1.489	13.30	0.59	23.01	0.85	1621	0.0398	0.29	0.2863	0.66	0.0521	0.58
NAN-8.6	252.911	0.192	257.501	0.740	15.80	0.65	13.36	0.28	2773	0.0400	0.08	0.2887	0.33	0.0524	0.29
NAN-8.7	252.027	0.283	251.971	0.618	0.10	0.65	35.23	0.30	6934	0.0399	0.11	0.2817	0.28	0.0513	0.18
NAN-8.9	252.932	0.108	255.945	0.699	11.09	0.66	8.44	0.19	2550	0.0400	0.04	0.2867	0.31	0.0520	0.29
NAN-8.10	252.084	0.077	253.421	0.303	5.46	0.69	40.09	0.20	11514	0.0399	0.03	0.2835	0.14	0.0516	0.09
NAN-8.11	251.983	0.152	252.107	0.441	0.83	0.71	27.98	0.38	4279	0.0398	0.06	0.2818	0.20	0.0513	0.18
NAN-8.12	253.427	0.594	253.353	1.253	0.01	0.78	6.56	0.20	1890	0.0401	0.24	0.2834	0.56	0.0513	0.48

a Isotopic dates calculated using the decay constants λ238 = 1.55125E-10 and λ235 = 9.8485E-10 (12).

b % discordance = 100 - (100 * (206Pb/238U date) / (207Pb/206Pb date)).

c Th contents calculated from radiogenic 208Pb and the 207Pb/206Pb date of the sample, assuming concordance between U-Th and Pb systems.

d Total mass of radiogenic Pb.

e Total mass of common Pb.

f Measured ratio corrected for fractionation and spike contribution only.

g Measured ratio corrected for fractionation, tracer and blank.

°° Corrected for initial Th/U disequilibrium using radiogenic 208Pb and Th/U_{magma} = 3.00.

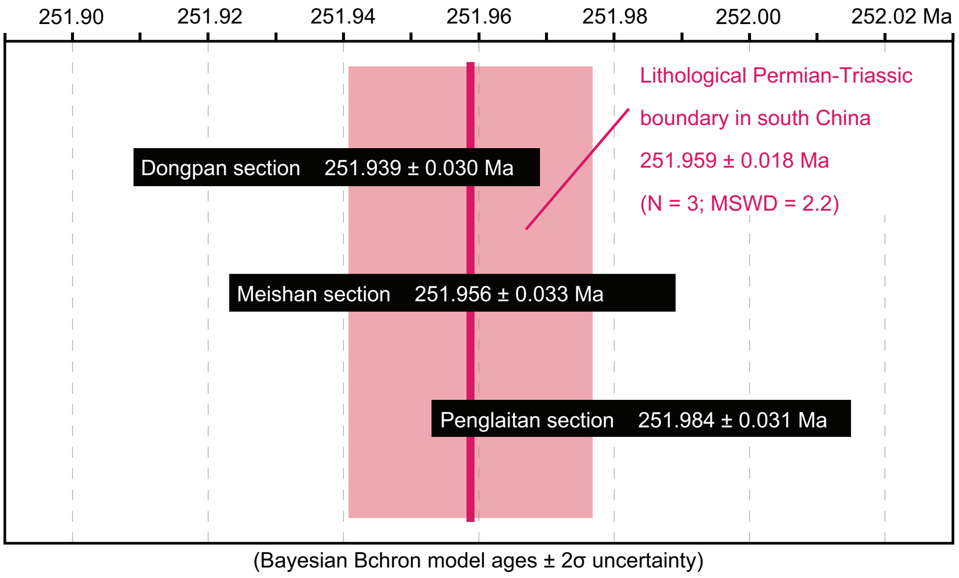

Figure S2. Calculated weighted mean age (251.959 ± 0.018 Ma; shown in pink) for the lithological Permian-Triassic boundary in China inferred from the Dongpan, Meishan and Penglaitan sections. The Bayesian Bchron model ages and their associated 2σ uncertainties (indicated by the black horizontal bars) for the lithological boundaries in Dongpan (251.939 ± 0.030 Ma), in Penglaitan (251.984 ± 0.031 Ma) and in the Meishan Global Stratotype Section and Point (251.956 ± 0.033 Ma) are taken from ref. 13 and are based on $^{206}Pb/^{238}U$ weighted mean zircon population dates from refs. 13,14.

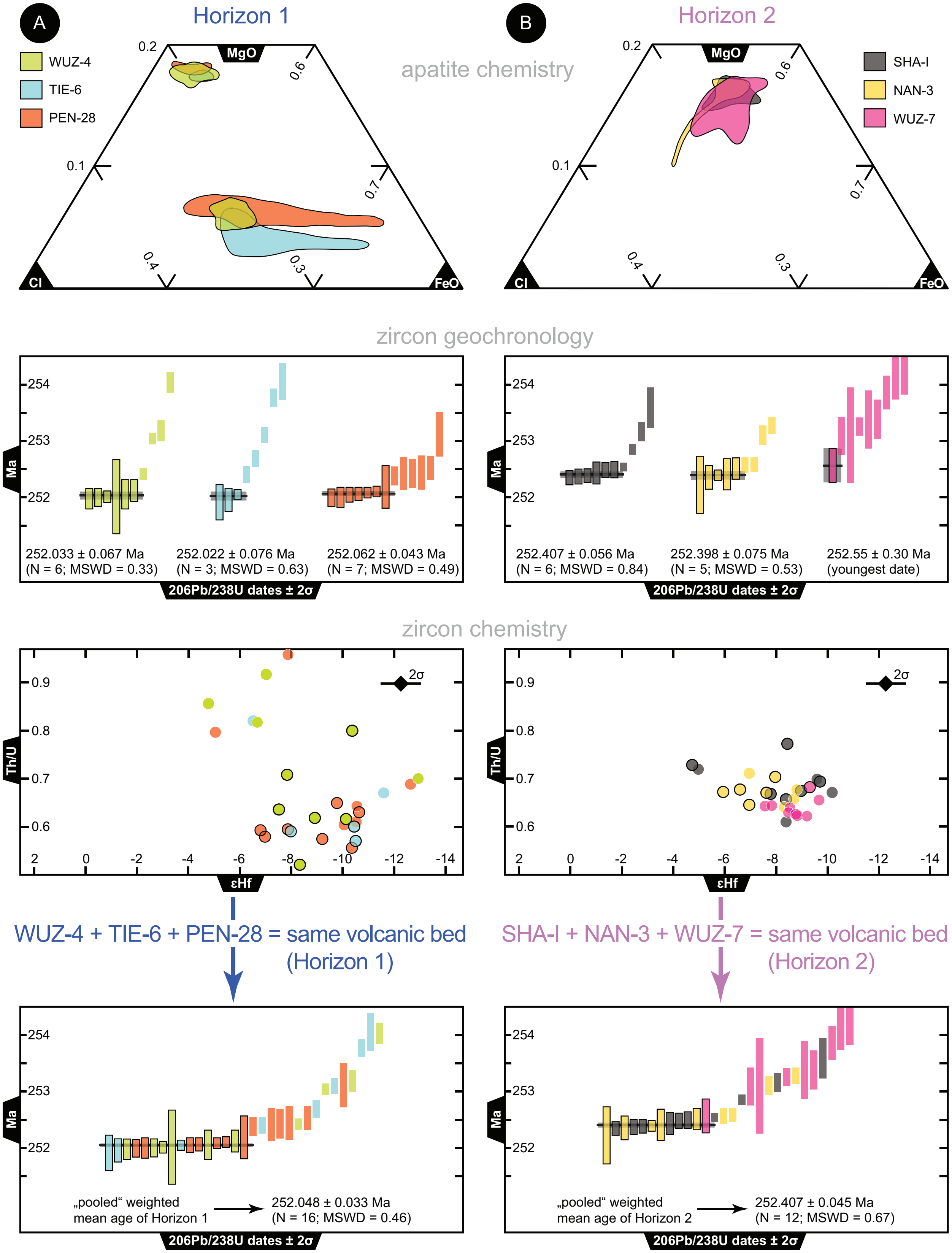

A
Horizon 1
WUZ-4
TIE-6
PEN-28
MgO
Cl
FeO
B
Horizon 2
SHA-I
NAN-3
WUZ-7
apatite chemistry
zircon geochronology
252.033 ± 0.067 Ma (N = 6; MSWD = 0.33)
252.022 ± 0.076 Ma (N = 3; MSWD = 0.63)
252.062 ± 0.043 Ma (N = 7; MSWD = 0.49)
252.407 ± 0.056 Ma (N = 6; MSWD = 0.84)
252.398 ± 0.075 Ma (N = 5; MSWD = 0.53)
252.55 ± 0.30 Ma (youngest date)
206Pb/238U dates ± 2σ
zircon chemistry
Th/U
εHf
2σ
WUZ-4 + TIE-6 + PEN-28 = same volcanic bed (Horizon 1)
SHA-I + NAN-3 + WUZ-7 = same volcanic bed (Horizon 2)
„pooled“ weighted mean age of Horizon 1
252.048 ± 0.033 Ma (N = 16; MSWD = 0.46)
„pooled“ weighted mean age of Horizon 2
252.407 ± 0.045 Ma (N = 12; MSWD = 0.67)

704

Figure S3. Apatite Cl-MgO-FeO ternary plots, zircon U-Pb ages, zircon Th/U versus εHf plots, and “pooled” $^{206}Pb/^{238}U$ weighted mean zircon population ages for Horizon 1 (A) and Horizon 2 (B) from ref. 15. Data reveal equality of correlated volcanogenic beds pooled in both horizons and reflect origin from the same volcanic eruption. A) Correlation of last Permian bed (Horizon 1) in Wuzhuan (WUZ-4), Tienbao (TIE-6), and Penglaitan (PEN-28). B) Correlation of Early Triassic volcanogenic sandstone bed (Horizon 2), which marks the top of the microbial limestone in the shallow-marine Shanmenhai (SHA-I), Nanem (NAN-3), and Wuzhuan (WUZ-7) sections. External reproducibility of Hf isotope analyses of 0.78 εHf (2σ) corresponds to reproducibility of Plešovice reference zircon measurements (15). MSWD = mean square of weighted deviates.

715

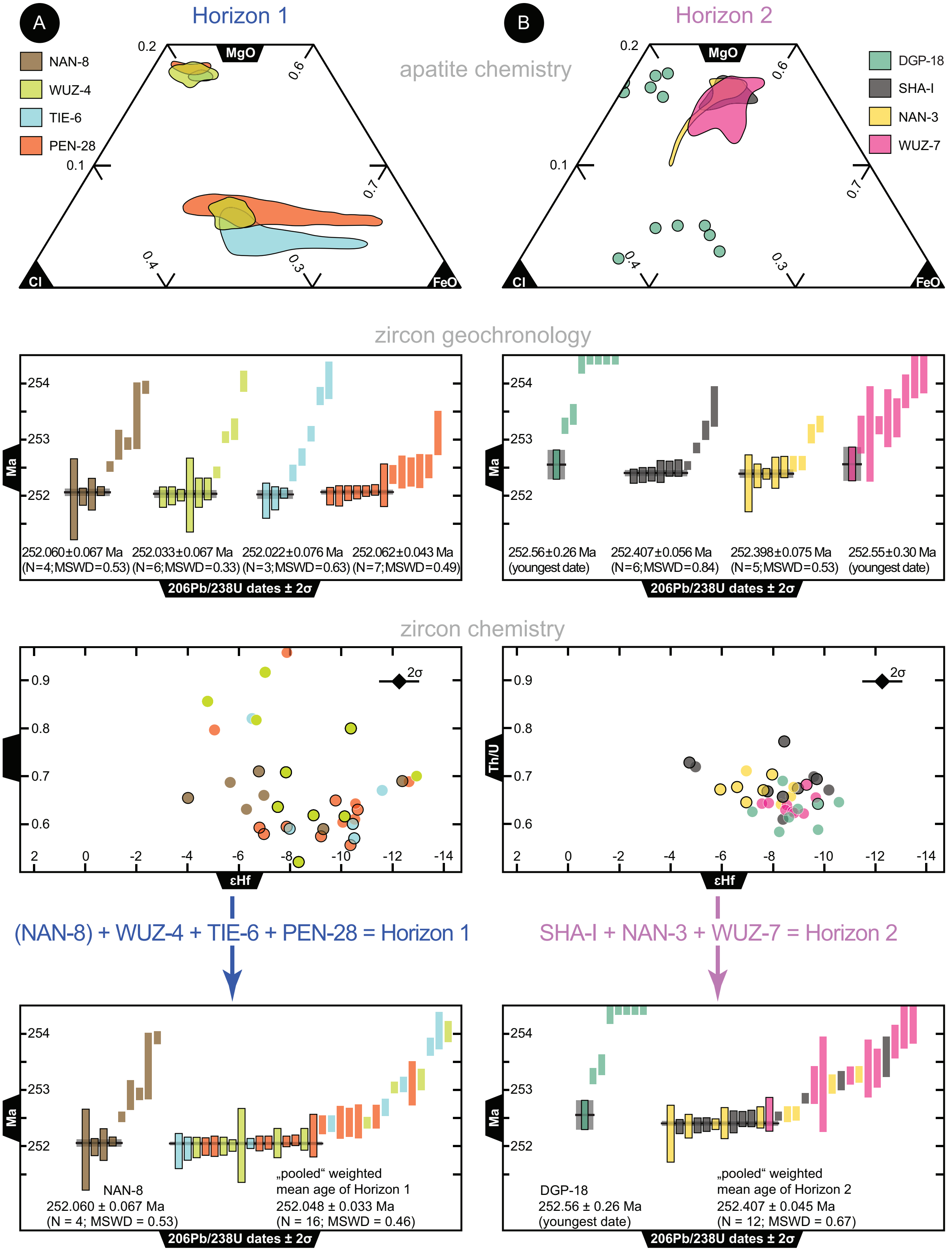
A
Horizon 1
B
Horizon 2
apatite chemistry
NAN-8
WUZ-4
TIE-6
PEN-28
DGP-18
SHA-I
NAN-3
WUZ-7
MgO
Cl
FeO
zircon geochronology
252.060±0.067 Ma (N=4;MSWD=0.53)
252.033±0.067 Ma (N=6;MSWD=0.33)
252.022±0.076 Ma (N=3;MSWD=0.63)
252.062±0.043 Ma (N=7;MSWD=0.49)
252.56±0.26 Ma (youngest date)
252.407±0.056 Ma (N=6;MSWD=0.84)
252.398±0.075 Ma (N=5;MSWD=0.53)
252.55±0.30 Ma (youngest date)
206Pb/238U dates ± 2σ
Ma
zircon chemistry
Th/U
εHf
2σ
(NAN-8) + WUZ-4 + TIE-6 + PEN-28 = Horizon 1
SHA-I + NAN-3 + WUZ-7 = Horizon 2
NAN-8
252.060 ± 0.067 Ma
(N = 4; MSWD = 0.53)
„pooled“ weighted mean age of Horizon 1
252.048 ± 0.033 Ma
(N = 16; MSWD = 0.46)
DGP-18
252.56 ± 0.26 Ma
(youngest date)
„pooled“ weighted mean age of Horizon 2
252.407 ± 0.045 Ma
(N = 12; MSWD = 0.67)

Figure S4. Apatite Cl-MgO-FeO ternary plots, zircon U-Pb ages, zircon Th/U versus εHf plots, and "pooled" $^{206}Pb/^{238}U$ weighted mean zircon population ages for Horizon 1 (A) and Horizon 2 (B) from ref. 15. Pooled volcanic beds in Horizon 1 and Horizon 2 reflect origin from the same volcanic eruptions, respectively. A) Correlation of last Permian bed (Horizon 1) in Wuzhuan (WUZ-4), Tienbao (TIE-6), and Penglaitan (PEN-28). NAN-8 shows similar zircon chemistry and identical $^{206}Pb/^{238}U$ weighted mean age as Horizon 1, but equality in apatite chemistry can not be tested due to the lack of apatite in NAN-8. B) Correlation of Early Triassic volcanogenic sandstone bed (Horizon 2), which marks the top of the microbial limestone in the shallow-marine Shanmenhai (SHA-I), Nanem (NAN-3), and Wuzhuan (WUZ-7) sections. A similar volcanogenic sandstone (DGP-18; 0.5 m above the lithological Permian-Triassic boundary) in the deeper marine Dongpan section shows identical zircon age spectra and chemistry as Horizon 2, but apatite chemistry reveals large spread in F, Cl, Fe and Mg composition. This either reflects different apatite composition than Horizon 2 and precludes origin from the same volcanic eruption, or might indicate alteration of the primary apatite composition. External reproducibility of Hf isotope analyses of 0.78 εHf (2σ) corresponds to reproducibility of Plešovice reference zircon measurements (15). MSWD = mean square of weighted deviates.

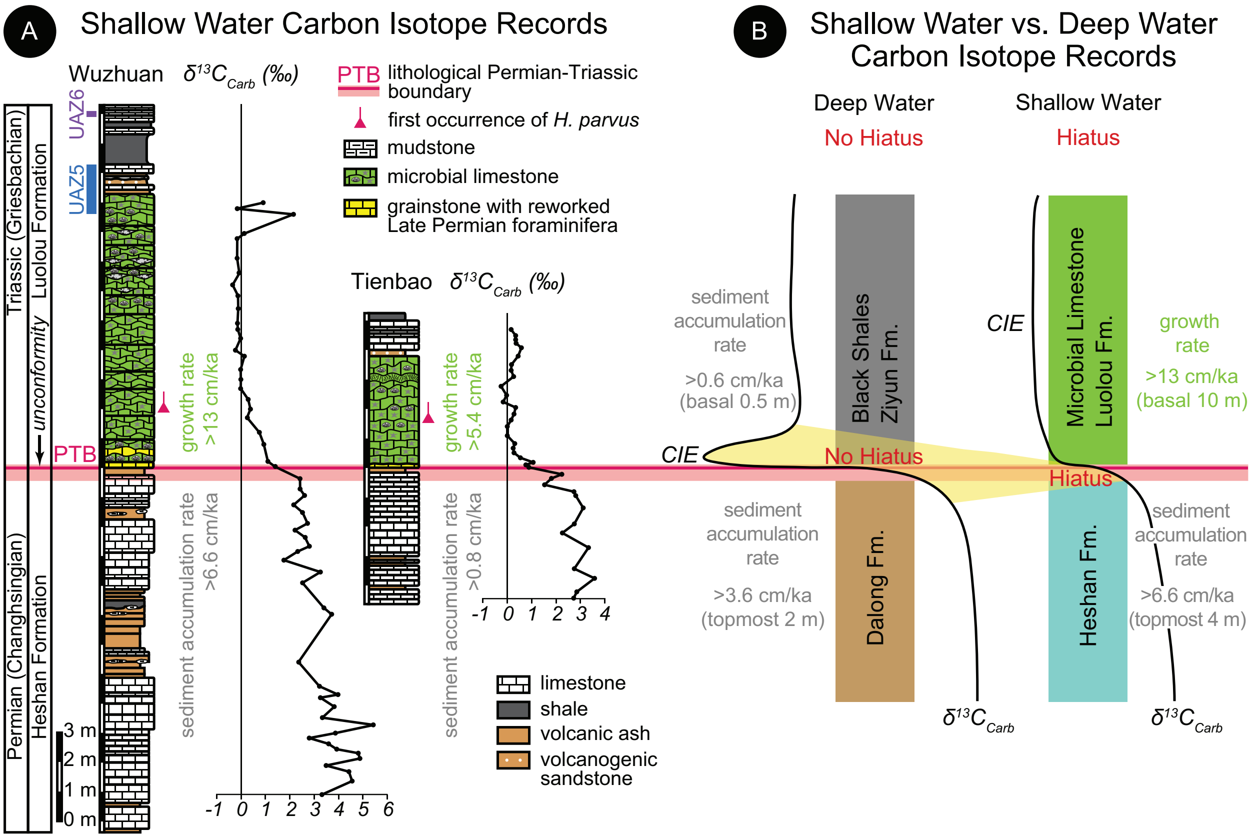

Figure S5. A) Carbonate carbon isotope chemostratigraphy of Wuzhuan and Tienbao (16) shows distinct negative excursions (drop from +4‰ to 0‰) starting at the Permian-Triassic boundary (PTB). Stratigraphic positions of the first occurrence of *Hindeodus parvus* and the durations of the Triassic conodont Unitary Association Zones UAZ5 and UAZ6 (17) are also indicated. Minimum sediment accumulation rates are calculated from U-Pb dates of the volcanic beds. B) Simplified model of the carbonate carbon isotope curves in deep and shallow water settings across the PTB in the Nanpanjiang Basin. Minimum sediment accumulation rates of deep water troughs of the Nanpanjiang Basin are generally lower and show a six-fold decrease from 3.6 cm/ka to 0.6 cm/ka across the PTB. Higher sediment accumulation rates in shallow water sections produce more expanded carbon isotope curves and stretched negative carbon isotope excursions (CIE). The hiatus in shallow water sections erased parts of the negative CIE, which is complete but compressed in the deep water sections (drop from +2‰ to -4‰).

References

1 Mattinson, J.M. Zircon U-Pb chemical abrasion ("CA-TIMS") method: Combined annealing and multi-step partial dissolution analysis for improved precision and accuracy of zircon ages. *Chemical Geology* **220**, 47-66, doi:10.1016/j.chemgeo.2005.03.011 (2005).

2 Condon, D.J. *et al.* Metrology and traceability of U-Pb isotope dilution geochronology (EARTHTIME Tracer Calibration Part I). *Geochimica et Cosmochimica Acta* **164**, 464-480, doi:10.1016/j.gca.2015.05.026 (2015).

3 Krogh, T.E. A low contamination method for hydrothermal decomposition of zircon and extraction of U and Pb for isotopic age determination. *Geochimica et Cosmochimica Acta* **37**, 485-494, doi:10.1016/0016-7037(73)90213-5 (1973).

4 Gerstenberger, H. & Haase, G. A highly effective emitter substance for mass spectrometric Pb isotope ratio determination. *Chemical Geology* **136**, 309-312, doi:10.1016/S0009-2541(96)00033-2 (1997).

5 Hiess, J., Condon, D.J., McLean, N.M. & Noble, S.R. $^{238}U/^{235}U$ Systematics in terrestrial uranium-bearing minerals. *Science* **335**, 1610-1614, doi:10.1126/science.1215507 (2012).

6 Bowring, J.F., McLean, N.M. & Bowring, S.A. Engineering cyber infrastructure for U-Pb geochronology: Tripoli and U-Pb_Redux. *Geochemistry, Geophysics, Geosystems* **12**, Q0AA19, doi:10.1029/2010GC003479 (2011).

7 McLean, N.M., Bowring, J.F. & Bowring, S.A. An algorithm for U-Pb isotope dilution data reduction and uncertainty propagation. *Geochemistry, Geophysics, Geosystems* **12**, Q0AA18, doi:10.1029/2010GC003478 (2011).

8 Bowring, S.A. *et al.* U/Pb zircon geochronology and tempo of the end-Permian mass extinction. *Science* **280**, 1039-1045, doi:10.1126/science.280.5366.1039 (1998).

9 Schoene, B. *et al.* Reassessing the uranium decay constants for geochronology using ID-TIMS U-Pb data. *Geochimica et Cosmochimica Acta* **70**, 426-445, doi:10.1016/j.gca.2005.09.007 (2006).

10 Renne, P.R. *et al.* Joint determination of ^{40}K decay constants and $^{40}Ar^{*}/^{40}K$ for the Fish Canyon sanidine standard, and improved accuracy for $^{40}Ar/^{39}Ar$ geochronology. *Geochimica et Cosmochimica Acta* **74**, 5349-5367, doi:10.1016/j.gca.2010.06.017 (2010).

11 Costa, F. Residence times of silicic magmas associated with calderas. Developments in *Volcanology* **10**, 1-55, doi:10.1016/S1871-644X(07)00001-0 (2008).

12 Jaffey, A.H. *et al.* Precision measurements of half-lives and specific activities of ^{235}U and ^{238}U. *Physical Review C* **4**, 1889-1906, doi:10.1103/physrevc.4.1889 (1971).

13 Baresel, B. *et al.* Precise age for the Permian-Triassic boundary in South China from high precision U-Pb geochronology and Bayesian age-depth modelling. *Solid Earth Discussions*, doi:10.5194/se-2016-145 (2016).

14 Burgess, S.D., Bowring, S.A. & Shen, S.Z. High-precision timeline for Earth's most severe extinction. *Proceedings of the National Academy of Sciences of the United States of America* **111**, 3316-3321, doi:10.1073/pnas.1317692111 (2014).

15 Baresel, B., D'Abzac, F.-X., Bucher, H. & Schaltegger, U. High-precision time-space correlation through coupled apatite and zircon tephrochronology: An example from the Permian-Triassic boundary in South China. *Geology* **45**, 83-86, doi:10.1130/g38181.1 (2016).

16 Bagherpour, B. *et al.* Onset, development, and cessation of basal Early Triassic microbialite in the Nanpanjiang pull-apart Basin, South China Block. *Gondwana Research*, in press, doi:10.1016/j.gr.2016.11.013 (2016).

17 Brosse, M., Bucher, H. & Goudemand, N. Quantitative biochronology of the Permian-Triassic boundary in South China based on conodont Unitary Associations. *Earth-Science Reviews* **155**, 153-171, doi:10.1016/j.earscirev.2016.02.003 (2016).
